# Using Telehealth-Delivered Procedures to Collect a Parent-Implemented Expressive Language Sampling Narrative Task in Monolingual and Bilingual Families With Autism Spectrum Disorder: A Pilot Study

**DOI:** 10.3389/fresc.2021.716550

**Published:** 2021-11-18

**Authors:** Laura del Hoyo Soriano, Lauren Bullard, Cesar Hoyos Alvarez, Angela John Thurman, Leonard Abbeduto

**Affiliations:** ^1^Department of Psychiatry and Behavioral Sciences, MIND Institute, University of California, Davis, Sacramento, CA, United States; ^2^Department of Spanish and Portuguese, University of California, Davis, Davis, CA, United States

**Keywords:** ASD, expressive language sampling, telehealth, bilingualism, parent-implemented

## Abstract

Language impairments are frequent, severe, and of prognostic value in autism spectrum disorder (ASD). Unfortunately, the evaluation of the efficacy of treatments targeting the language skills of those with ASD continues to be hindered by a lack of psychometrically sound outcome measures. Expressive Language Sampling (ELS) procedures offer a promising alternative to norm-referenced standardized tests for assessing expressive language in treatment studies. Until now, however, research on the validity and utility of ELS as outcome measures has been limited to administrations by a trained professional in a clinic setting and to use with English-speaking families. These limitations are a barrier for many families accessing the benefits of participation in treatment studies. The current study examines the feasibility of teaching native English-speaking parents (NESP) and native Spanish-speaking parents (NSSP) how to administer the ELS narrative task (ELS-N) to their sons and daughters with ASD (between ages 6 and 21) at home through telehealth-delivered procedures. The parent training was provided in the primary language of the participating parent (i.e., 11 NSSP and 11 NESP) and administered by the parent to the youth in the language that the parent reported to use to communicate with the youth at home (i.e., 9 Spanish and 13 English). Families were able to choose between using their own technology or be provided with the technology needed for participation. Of the 19 parents who completed the training, 16 learned to administer the ELS-N procedures. In addition, strong test-retest reliability and no practice effects over the 4-week interval were observed for ELS-N derived youth outcome measures (i.e., talkativeness, vocabulary, syntax, dysfluency, and intelligibility) for both NSSP and NESP. Results from this pilot study suggest that the home-based parent-implemented ELS-N procedures can be learned and administered at acceptable levels of fidelity by parents, with good test-retest reliability and limited practice effects observed in terms of outcome measures for youth with ASD. Implications for treatment studies and future directions are discussed.

## Introduction

The prevalence of autism spectrum disorder (ASD) has drastically increased in the recent decades (1), with the most recent estimate being 1 in 54 children identified with ASD (2). As this prevalence has increased, so has an interest in developing behavioral and pharmacological treatments for both core symptoms (i.e., limitations in social communication as well as restricted and repetitive behaviors) and co-occurring challenges (3). In terms of co-occurring challenges, individuals with ASD have varying degrees of expressive language difficulties, which can include difficulties in phonological skills (4) as well as delays in lexical, syntactic, pragmatic, and semantic skills (5–9). These language difficulties are more severe than deficits in other cognitive domains for many individuals with ASD (10–14). In addition, expressive language skills in early childhood are one of the best predictors of adult outcomes in terms of independent living and adaptive functioning for the ASD population (15, 16). Expressive language, therefore, is often directly or indirectly, a treatment target for individuals with ASD. There is a need, however, for psychometrically sound measures to assess expressive language to determine treatment and developmental progress (17).

Although many norm-referenced standardized tests for assessing expressive language are available, these are poorly suited for use in treatment studies on individuals with ASD and other neurodevelopmental conditions (18). One of the greatest challenges encountered by treatment studies in ASD is measuring improvements in heterogeneous samples. Individuals with ASD demonstrate both a wide range of language abilities, as well as a variability in their performance across language domains. As a result, the composite scores that are typically provided through norm-referenced tests may mask improvements and not all individuals with ASD can manage the demands of standardized tests. Furthermore, most norm-referenced tests rely on formats that are unlike real-world language use and thus, their generalizability to everyday language use is often limited.

Expressive language sampling procedures (ELS) are an attractive alternative to norm-referenced standardized tests (18). ELS procedures entail collecting and analyzing relatively brief samples of spoken language in a naturalistic context in such a way that the language sample is representative of the individual's “everyday” language abilities. Narrative procedures (ELS-N), for example, involve a wordless picture book as a visual stimulus from which participants are prompted to engage in storytelling with minimal scaffolding provided by a professional examiner. Narrative procedures are particularly appealing for use in ASD and other neurodevelopmental conditions because they can be used in wide age ranges and ability levels. Indeed, (1) storytelling is developmentally appropriate from early childhood into adulthood, (2) picture books can be selected in ways that are appropriate for a wide range of ages, and (3) narrative events can be described in concrete or abstract ways, with simple or complex syntax, etc. (19, 20). In addition, the ELS-N procedures can distinguish between language profiles on specific neurodevelopmental populations (21) and capture change over time (22). For all these reasons, the ELS-N task has been proposed as a potential outcome measure for clinical trials involving individuals with intellectual and developmental disabilities (23–25) and has been used in several clinical trials for these populations (e.g., Protocol Neu-2566-FXS-001; A Safety Study of NNZ-2566 in Patients With Fragile X Syndrome, 2013–2017). In terms of its psychometrical proprieties, the ELS-N procedures administered by a professional examiner (e.g., clinician) to individuals with fragile X syndrome (FXS) or Down syndrome (DS) have been associated with very low rates of non-compliance, evidence minimal practice effects, are largely free of floor and ceiling effects, are highly reliable over repeated administrations to the same participant, and demonstrate strong construct validity (26, 27). In addition, ELS-N procedures have several advantages relative to norm-referenced standardized tests, such as allowing the computation of a wide range of dependent variables (e.g., from pragmatics to syntax), being more closely aligned with real-world performance, and being more generalizable to functional activities (28). Note that both DS and FXS are associated with higher rates of co-occurring ASD than is the general population, with FXS in particular sharing many phenotypic similarities with ASD (29–31). These phenotypic similarities suggest that ELS-N might be a promising outcome measure for those with ASD as well. Indeed, Barokova et al. (32) recently provided preliminary evidence that ELS-N procedures developed to assess minimally verbal individuals with ASD yielded measures with strong psychometric properties (32).

In the current study, we extended our previous work on the ELS-N procedures by addressing three important limitations. First, like many other outcome measures, the ELS-N procedures require an in-person administration by a trained examiner, typically in a clinic or research setting. Because treatment studies usually require multiple administrations of the outcome measures and thus, many trips to the clinic, this is a burden that is costly to the family and investigator and discourages many families from enrolling and contributing to drop out, especially for families located in rural areas. Second, the ELS-N procedures that have been psychometrically evaluated for ASD, FXS, and DS only exist in an English language version. This limitation is particularly problematic considering the rapidly growing Spanish-speaking population in the U.S. (33). Third, many individuals with ASD may have increased non-compliance when being assessed by an unfamiliar examiner because of an inability to adapt to new circumstances or co-occurring anxiety (34–36), thereby adversely affecting their expressive language performance during the assessment. These limitations effectively preclude families who cannot afford to travel, or whose children's behavior makes travel impossible, as well as non-English-speaking families, from accessing the potential benefits of participating in treatment studies and restricts the representation of these individuals within the scientific literature.

In the current study, we have extended the ELS-N procedures in two ways. First, we have created a set of telehealth-delivered training procedures that enable parents to learn and administer the ELS-N procedures to their children at home. Second, we have created a culturally and linguistically appropriate Spanish-language version of the ELS-N task and the parent training procedures. In the current pilot study, we present preliminary data on this new home-based parent-implemented version of ELS-N procedures in English and Spanish-speaking families of children and adolescents with ASD in order to address the following research questions:

(1) Can parents learn to administer the ELS-N task to their children with ASD to an acceptable level of fidelity?
(2) What are the psychometric proprieties of the ELS-N task in ASD when administered by parents from home?
(3) What are parental perspectives on the training procedures and the ELS-N task?

## Methods

### Participants

A total of 22 parent-child dyads participated in this project. Participants were recruited from the MIND Institute Volunteer Registry, through its UC Davis Health StudyPage system, and through the UC Davis MIND Institute Resource Center. Participating parents were between 26 and 56 years of age, 20 were females and 2 males. Eleven parents self-identified as native English-speaking parents (NESP) and the other 11 as native Spanish-speaking parents (NSSP). All NESP indicated that they were monolingual, whereas all NSSP indicated that they were bilingual, with English as their second language. Participants with ASD were between 6 and 21 years of age, with 17 males and 5 females. The 11 NESP reported that their sons/daughters were monolingual English-speakers, 2 NSSP reported that their sons/daughters were monolingual English-speakers, and the other 9 NSSP reported that their sons/daughters were bilingual (Spanish-English).

Inclusion criteria for the youth were: (1) a community-based ASD diagnosis confirmed with a physician or school report shared by parents, (2) chronological age between 6 and 23.99 years of age at the start of study participation, (3) no other comorbid neurodevelopmental disorders (e.g., FXS or DS), (4) able to produce at least two-word utterances on occasion (according to parent eligibility interview), (5) must live at home and have caregivers who make medical decisions on their behalf, and (6) can assent to participate. Inclusion criteria for the parents were: (1) older than 18 years of age, (2) able to consent to participate, and (3) able to provide a copy of the clinical report confirming the ASD diagnosis of the youth. Note that both parents and participants with ASD did not (according to parent report) have uncorrected sensory or motor impairments that would preclude participation. Lastly, English or Spanish was the self-reported primary language of all the participants consistent with group assignment. See Table 1 for characteristics of the parents and youth. At the time of recruitment, parents were also queried about the language levels of the children so as to determine that the children had sufficient language skills to meaningfully complete the ELS-N task. In particular, parents were asked: *What is the primary form of communication for your child?* If speech, we then asked parents to provide an example of something that the child said yesterday or today and how the child would describe a wordless picture image in which there is a frog jumping into a boy's pocket. Based on the parental responses, youth with ASD were classified into the following mutually exclusive categories: (a) participant produces 2-word utterances on occasion, (b) participant produces 3- to 4-word utterances on occasion, (c) participant produces ≥4-word utterances daily. The distribution of participants into these categories is presented in Table 1. As seen in Table 1, most youth fell into the most mature of the three categories.

**Table 1:** Demographics and language-related characteristics of participants.

**NSSP group (*n* = 11)**	**NSSP group (*n* = 11)**	**NESSP group (*n* = 11)**
Caregiver race	11 Latinx	10 White 1 Black or African American
Caregiver age	M = 42; SD = 8.4; R = 26–55	M = 43.4; SD = 6.8; R = 34–56
Caregiver sex	10 Females, 1 Male	10 Females, 1 Male
Caregiver primary language	11 Spanish	11 English
Caregiver other languages^*^	11 bilinguals (Spanish/English)	11 monolinguals (English)
Caregiver education	1 Kindergarten−8th Grade (some or all) 3 Graduated Highschool/GED 1 Some college or technical school 1 Graduated: Associate's or technical degree 1 Graduated: Master's/Other graduate degree 4 missing	2 Some college or technical school 3 Graduated: Bachelor's degree 3 Graduated: Master's/Other graduate degree 3 missing
Household income	1 $ 15.001–20.000 1 $ 45.001–50.000 1 $ 50.001–60.000 3 $ 60.001–70.000 1 I don't know 4 missing	1 $ 40.001–45.000 1 $ 60.001–70.000 1 $ 90.001–100.000 1 $ 100.001–150.000 2 $ 150.001–200.000 2 $ More than 300.000 3 missing
Child race	11 Latinx	10 White 1 Black or African American
Child age	M = 10.6; SD = 5.5; R = 6–20	M = 10.3; SD = 4.3; R = 6–21
Child sex	4 Females, 7 Males	1 Females, 10 Males
Child primary language^*^	5 equal use of Spanish/English 4 preferred English 2 only English	11 only English
Child bilingualism^*^	9 bilinguals (Spanish/English) 2 monolinguals (English)	11 monolinguals (English)
Level of child's expressive language^*^	0 produce 2-word utterances on occasion 2 produce 3-to-4-word utterance on occasion 9 produce >4-word utterances daily	1 produce 2-word utterances on occasion 0 produce 3-to-4-word utterance on occasion 10 produce >4-word utterances daily

* *As reported by the parent in the screening interview; M, mean; SD, standard deviation; R, range; NSSP, native Spanish-speaking parents; NESP, native English-speaking parents*.

### Procedures and Measures

We first conducted a focus group of 3 females and 1 male between 39 and 50 years of age, all parents of individuals with ASD who not only self-identified as Spanish-English bilinguals, but also view themselves as native Spanish speakers and reported that English was their second language. The purpose of the focus group was to discuss the rationale and procedures for training parents in the ELS-N administration, as well as the materials to be used during the administration of the task. Therefore, the ultimate purpose of the focus group was to gain insight into any aspects of the procedures that needed to be adjusted to ensure language and cultural appropriateness and acceptability. We then incorporated the insights from the focus group into the final set of materials and procedures described in the present report. The study was approved by the Institutional Review Board of the University of California, Davis. The authors assert that all procedures contributing to this work complied with the ethical standards of the relevant national and institutional committees on human experimentation and with the Helsinki Declaration of 1975, as revised in 2008. Written informed consent from all parents and verbal assent from all participants with ASD was obtained before their participation in the study.

#### Materials

The following materials were provided to the participating parents to support their learning of the procedures A technology setup guide, a parent-friendly version of the original ELS-N administration manual developed by Abbeduto et al. (26), a PowerPoint^TM^ presentation regarding the ELS-N principles, video examples of in-clinic ELS-N administrations by clinicians and researchers, and parent-friendly versions of the ELS-N scripts to be used during the administration of the task. The technology materials needed for participation were a laptop computer (e.g., MacBook Air) equipped with a web camera, an earpiece device, and an iPad containing the complete set of wordless picture books to be used during the ELS-N administrations. Parents were offered a choice between using their own technology or being sent all or part of the technological materials needed. One family preferred to use their own laptop, with the remainder of the technological materials provided to the parent. All other families were provided all the materials needed for study participation. Office 365 SharePoint^TM^ was used for study-related communication (e.g., joining Skype for Business^TM^) as well as to safely transfer coded media files in accordance with UC Davis Health Systems IT policies. All the training materials were provided in the primary language of the parent as reported by parents (i.e., 11 NSSP and 11 NESP). The only exception to this was that for the 2 NSSPs who indicated that their children were monolingual English-speakers, and therefore would administer the task in English. For these two parents, the ELS-N scripts to be used during the ELS-N administration were provided in English, with the remaining training materials provided in Spanish.

#### The ELS-N Task

The original ELS-N task is designed to elicit expressive language from a participant using different wordless picture books and with a minimum amount of participation and scaffolding provided by the examiner. After an initial silent viewing of the book to get a sense of the story, the participant is prompted by the examiner to tell the story page by page. Page-turning is timed and controlled by the examiner who follows a script regarding the procedure directions, which include a set of prompts to be used depending on the child's language input and behavior, as well as the time wait for each procedure. Administration is untimed, but the typical range is 12–15 min (26, 27).

In the present study, the goal for parents was to administer the ELS-N as a trained examiner would, with the only exception being a modest adjustment of the fidelity standards for parents relative to professionals. Such adjustment seemed a reasonable accommodation given that parents are not familiar with neuropsychological assessment principles in contrast to a professional examiner. The achievement of fidelity standards was evaluated by a staff member through a fidelity scoring rubric (FSR), which assesses the adequacy of the prompts to be used by the parent during the ELS-N administration as well as other aspects of the protocol (e.g., position the book so the child can see it, time wait for each page). Administration procedures are assessed on a one-point basis. For example, if during the assessment an administration prompt isn't used or is used incorrectly (e.g., too early, in the wrong page or unnecessarily used) a point is deducted from the rubric. The final score is a percentage which represents the level of proficiency in which the examiner administers the ELS-N task. An 80 % on the FSR was considered the fidelity standard in this study. Note that all the procedures for the examiner-administered version (e.g., the ELS-N administration manual, the ELS-N scripts, and the ELS-N fidelity FSR) are available online at https://ctscassist.ucdmc.ucdavis.edu/ctscassist/surveys/?s=W9W99JLMNX. The materials adapted for families used in this pilot project are also available upon reasonable request from the corresponding author.

The ELS-N administrations were videorecorded by the examiner (the parent). Then, these recordings were transcribed by highly trained assistants following transcription procedures developed previously (26) and analyzed using computer-based algorithms to derive clinical endpoints reflecting several dimensions of the language ability of the participant with ASD (37). The following five language variables were derived from transcripts segmented into C-units (i.e., an independent clause and its modifiers): number of C-units attempted per minute (talkativeness), number of different word roots in up to 50 C-units (lexical diversity), C-unit length in morphemes (syntactic complexity), percentage of C-units containing dysfluency (planning), and percentage of C-units that were fully or partly unintelligible (unintelligibility). We also evaluated the extent to which the participants with ASD meaningfully completed the ELS-N procedures as an indication of feasibility: (a) transcribers noted whether a youth with ASD was compliant, with non-compliance defined as explicit refusal to do the task (e.g., saying, “I'm done”) or repeatedly engaging in off-task behavior and (b) samples with less than “one task-relevant C-unit on every page of the book were not considered complete samples in the present study.

#### Parent Training

The purpose of the training was to teach parents to administer the ELS-N task to the predetermined level of fidelity described below (see sections: Homework Block and Test-Retest Administrations). The training for the NSSP group was provided by a Spanish-English bilingual staff member for whom Spanish was her native and primary language, and the training for NESP group was provided by a monolingual staff member for whom English was her native language. The entire training process for each parent lasted a total of 9–10 weeks and was divided into the sessions described below. Note that a different wordless picture book from the Mercer Mayer's “frog” series was used across each administration for each participant.

*Technology Set Up:* The first session of the parent training involved a review of the technology set-up guide. The session started with a regular phone call during which parents were guided in how to log into a computer, access their unique SharePoint ^TM^ site, and join the secure Skype for Business ^TM^ video call. Once the participating parent successfully connects to the video-teleconferencing call, parents then learn how to videorecord their ELS-N administrations and upload the video to their SharePoint ^TM^ site by practicing in real-time (e.g., videorecord themselves and uploading the video) while connected with the research staff member to make sure that the parent understands each technology procedure. During this session, parents were also asked to log-into the iPad and access the wordless picture books that will be used during each session. The session lasted 15–45 min, depending on the family.

*Initial Training:* After the technology setup session, the initial training session for the ELS-N was conducted. In this 1-h session, the research staff member reviewed with the parent the instructions on how to administer the ELS-N using the PowerPoint ^TM^ presentation and video examples of ELS-N administration by an examiner. The ELS-N administration manual, as well as the ELS-N scripts for administration and the digitalized wordless picture books, were reviewed in this session.

*Coaching:* After the initial training session, the coaching session was conducted. In this 30-min session, the parent was asked to administer the ELS-N task to his/her son/daughter. The research staff member observed the session live over Skype for Business^TM^ and offered coaching suggestions to the parent in real-time through an earpiece worn by the parent.

*Homework Block:* Following the coaching session, parents were asked to practice and video-record at least three administrations of the ELS-N using different wordless picture books for each administration over a period of 2–4 weeks. The parent uploaded the video-recordings to their SharePoint ^TM^ site, with the administration evaluated by a research staff member against the FSR. Although the fidelity standards were based on an 80% on the FSR, in order to achieve proficiency during homework (i.e., parent is ready for test-retest), they needed to obtain one score of at least 90% correct according to the FSR on one ELS-N administration and at least 80% correct according to the FSR on a second. The FSR is not shared with the participating parent; instead, the research staff member provides written substantive feedback *via* email, citing all deviations from the gold-standard administration embodied in the FSR. The written feedback was provided in the primary language of the parent. The feedback was emailed to the parent the same day or the day after the practice session was uploaded and before the next homework session was conducted by the family. If the parent failed to achieve the proficiency criterion within the three homework sessions, extra homework sessions were required until the proficiency criterion was achieved or until a maximum of five homework sessions were conducted.

*Test-Retest Administrations:* Once parents completed the *Homework Block*, they conducted two more ELS-N administrations, with a target interval of 4 weeks between the two administrations. Although no feedback was provided to the parents on these test-retest administrations, the FSR was still applied to determine deviations from the gold-standard administration. In order to maintain fidelity during test-retest, parents needed to obtain at least 80% correct according to the FSR on both test-retest administrations. Note that only test and retest sessions were transcribed and coded to analyze youth language, and this was done following Abbeduto et al. (26) procedures.

*Exit interview:* Following the completion of the retest session, parents were interviewed in their primary language over Skype for Business ^TM^ and asked open-ended questions regarding the adequacy of training and the extent to which they would be willing to conduct the testing within the context of a clinical trial in lieu of traveling to a clinic and having a clinician complete the testing^1^. In addition, a question regarding the language and cultural appropriateness of the materials and training is also asked the NSSP group. The session lasted 10–30 min, depending on the family. See Table 2 for the complete list of questions asked to the parent on the exit interview. Also, see Table 3 for a summary of key components of each session of the parent training, including time, materials needed, participants involved, etc.

**Table 2:** Exit interview questions for parents.

1. Did you feel that the training materials were adequate for teaching you how to give the ELS-N to your son/daughter?
2. Can you tell which things you liked and which things need to improve?
3. Did you find the ELS-N easy or difficult to learn?
4. How confident are you that you learned how to give the test correctly?
5. If your son/daughter was in a treatment study, would you rather come to the clinic to participate or stay home and give the NLS test. Why?
6. Do you want to share anything you consider important, but I didn't ask you before?
7. The following question was for NSSP group only: Can you describe whether you think the training you received and the NLS test itself were appropriate for Spanish speakers and their culture?

**Table 3:** Key components of the parent training sessions.

**Sessions**	**Duration**	**Materials**	**Technology**	**Participants**	**Other details**
Technology set up	15–45 min	• Technology set up guide • Wordless picture books	• Laptop • Web camera • iPad • Earpiece device (only for coaching)	Parent and staff member	Synchronic communication between parent and staff member. Feedback to parent is verbally provided during the session.
Initial training	1 h	• ELS-N administration manual • PowerPoint ^TM^ presentation • Video examples of in clinic ELS-N admin. • Wordless picture books • Scripts			
Coaching	30 min	• Wordless picture books • Scripts		Parent, child, and staff member	
Homework block (3–5 sessions)	15–20 min		• Laptop • Web camera • iPad	Parent and child	• Non-synchronic communication between parent and staff member: • FSR applied. • Feedback against FSR is provided to parent via email.
Test-retest (2 sessions)			• Laptop • Web camera • iPad		• FSR applied. • Feedback against FSR is not provided to parent. • Language variables derived from the child.
Exit interview	10–30 min	Exit interview (for the staff member)	• Laptop • Web camera	Parent and staff member	Synchronic communication between parent and staff member.

### Statistical Analyses

The following analyses were conducted for each of our research questions. Because of the small sample size, we present data both before and after correcting for multiple analyses in each of these parametric analyses. Correction for conducting multiple tests was done using the Bonferroni correction to maintain a familywise alpha rate of *p* < 0.050.

#### Can Parents Learn to Administer the ELS-N Task to Their Children With ASD to an Acceptable Level of Fidelity?

To address this question, we computed descriptive statistics regarding parents' scores on the FSR during homework and test-retest administrations.

#### What Are the Psychometric Proprieties of the ELS-N in ASD When Administered by Parents From Home?

To address this question, we evaluated feasibility, practice effects, and test-retest reliability. First, to evaluate whether the procedures were feasible for children and adolescents with ASD, we computed descriptive statistics on how many youths were able to complete the narrative procedures in meaningful ways (i.e., feasibility). Second, to evaluate whether there were practice effects between test-retest administrations, we conducted *t*-tests for correlated samples for each of the five language variables for the youth. Third, to examine test-retest reliability, we computed Pearson and intraclass correlations between the two administrations for each of the five language variables for the youth. In computing the intraclass correlations, we reported results for a mixed model, assuming no interaction and absolute agreement. All these analyses were conducted separately for each language group (i.e., NSSP vs. NESP). In addition, tests were performed twice: First, including samples from all compliant participants with ASD regardless of parent score on the FSR and second, including samples from all compliant participants with ASD whose caregivers administered the ELS-N ≥ 80% on the FSR at both test and retest sessions.

#### What Are Parental Opinions of the Training and the ELS-N Task?

To address this question, we evaluated parental opinions regarding the training expressed during the exit interviews through qualitative analyses.

## Results

### Can Parents Learn to Administer the ELS-N Task to Their Children With ASD to an Acceptable Level of Fidelity?

Nineteen of the twenty-two parent-child dyads who participated in this project completed the training. Three parent-child dyads (i.e., 1 NSSP and 2 NESP) discontinued training for reasons unrelated to the study (e.g., family circumstances). The two parents from the NESP group who ended early nonetheless were able to administer the ELS-N at >90% on the FSR as early as their first homework session. The parent from the NSSP who withdrew from the study did so after her third homework session and she did not reach fidelity (i.e., >80% on the FSR) at any session. Note that all parents who completed the training, except for 1, were able to administer the ELS-N > 80% on the FSR in at least one ELS-N administration (see Table 4 for details).

**Table 4:** Percent of fidelity scoring rubric (FSR) elements scored correct in each session for the 22 parents who participated in the study.

**Parent ID**	**Homework 1**	**Homework 2**	**Homework 3**	**Homework 4**	**Homework 5**	**Test**	**Re-Test**
NSSP-1	37.7%	78.3%	52.2%	61.6%	59.6%^FNR^	65.7%	83.0%
NSSP-2	60.0%	84.3%	74.0%	83.0%	91.6%^5^	91.0%	96.6%
NSSP-3	68.0%	68.3%	92.6%	90.0%^4^	NN	96.4%	91.0%
NSSP-4	71.0%	83.0%	81.0%	87.0%	68.0%	95.0%	100.0%
NSSP-5	72.0%	89.8%	100.0%^3^	NN	NN	72.7%	91.0%
NSSP-6	73.4%	91.8%	88.4%^3^	NN	NN	90.9%	82.%
NSSP-7	43.8%	23.4%	38.0%	47.0%	54.6% ^FNR^	54.5%	28.4%
NSSP-8	94.7%	89.5%^2^	98.0%	NN	NN	100.0%	91.2%
NSSP-9	42.8%	68.4%	100%	98.2%^4^	NN	96.4%	95.5%
NSSP-10^D^	51.9%	40.0%	49.8%	–	–	–	–
NSSP-11	17.0%	41.7%	28.8%	55.2%	76.6% ^FNR^	83.1%	80.0%
NESP-1	71.0%	90.0%	86.0%^3^	NN	NN	94.0%	78.0%
NESP-2	73.0%	86.0%	93.0%^3^	NN	NN	85.0%	81.0%
NESP-3	82.0%	80.7%	91.0%^3^	NN	NN	95.5%	62.9%
NESP-4^D^	90.0%	–	–	–	–	–	–
NESP-5	96.0%	87.7%^2^	88.0%	NN	NN	98.2%	69.6%
NESP-6	72.0%	98.0%	88.0%^3^	NN	NN	91.8%	91.2%
NESP-7	78.1%	84.7%	88.0%	93.0%^4^	NN	96.7%	89.3%
NESP-8	88.0%	84.0%	90.7%^3^	NN	NN	82.7%	95.1%
NESP-9	92.0%	95.7%^2^	74.5%	NN	NN	96.6%	99.1%
NESP-10	97.3%	90.4%^2^	88.5%	NN	NN	98.2%	100.0%
NESP-11^D^	94.7%	92.9%^2^	98.0%	NN	NN	–	–

*Percentages correspond to the score achieved by each parent in the FRN for each ELS-N administration. Proficiency during homework was predetermined as achieving at least one FNR > 80 in one session + a > 90 in another session. In order to be cleared for Test-retest all parents were required to conduct at least 3 homework administrations even if they achieved proficiency in two sessions. The exponent number on the last homework session corresponds to the number of homework sessions in which parents reached proficiency (e.g., 2, 3, 4, or 5). Parents with both Test and Re-test shadowed are those who maintained fidelity (i.e., >80%) in both sessions. NSSP, native Spanish-speaking parents; NESP, native English-speaking parents; D, family dropped; –, sessions not completed due to family dropping; NN, sessions not needed, FNR, fidelity not reached*.

During the *Homework Block*, 15 (i.e., 6 NSSP and 9 NESP) of the 19 (i.e., 10 NSSP, 9 NESP) parents who completed the training were able to learn to administer the ELS-N procedures to the predetermined level of proficiency required to be cleared for test-retest administrations. Four parents met the designated proficiency in two sessions (i.e., 1 NSSP and 3 NESP), seven parents in three sessions (i.e., 2 NSSP and 5 NESP), three parents in four sessions (i.e., 2 NSSP and 1 NESP), and one parent from the NSSP group in five sessions.

Of the 15 parents who reached the designated proficiency during the *Homework Block*, four were not able to maintain fidelity at both test-retest administrations (i.e., 1 NSSP and 3 NESP). Interestingly, of the four parents who did not achieve the designated proficiency during the *homework block*, two were able to achieve and maintain fidelity in both test and retest administrations. As seen in Table 4, although 16 out of the 19 parents achieved fidelity in the test session (i.e., 7 NSSP and 9 NESP), only 13 of 19 parents maintained fidelity in both test and retest administrations (i.e., 7 NSSP and 6 NESP). Descriptive statistics regarding the percent of FSR elements scored correctly by the parents at each ELS-N administration are provided in Table 4.

### What Are the Psychometric Proprieties of the ELS-N in ASD When Administered by Parents From Home?

Note that all participants with ASD from the NESP group produced narrative samples entirely in English. In the NSSP groups, 4 of the 10 participants with ASD produced narrative samples combining Spanish and English, 2 others produced narrative samples entirely in Spanish, and the remaining 4 participants with ASD produced narrative samples entirely in English. As reported by caregivers, 2 of the 4 ASD participants who produced English narrative samples were bilingual (English/Spanish), whereas the remaining 2 were monolingual (English). For these last 2 participants, the parents learned the procedures in Spanish but administered the task to the youth in English [see this case report study by del Hoyo Soriano et al. (38) for further details].

#### Feasibility

One participant with ASD from the NSSP group produced an incomplete sample on both the test and retest administrations. This participant was the one whose father reported that she produces 2-word utterances on occasion during the screening interview (see Table 1). Another participant with ASD from the NESP group did not produce a complete sample for the test administration. This participant was one whose mother reported that he produces 3-to-4-word utterance on occasion during the screening interview (see Table 1). All other participants with ASD were compliant and produced complete samples in the test and retest administrations.

#### Practice Effects and Test-Retest Reliability

The data on the potential practice effects and reliability across the test and retest administrations of the ELS-N for each language group are presented in Table 5 (practice effects) and 6 (reliability).

**Table 5:** T-test for practice effects on test-retest administrations over the 4-week interval.

**Expressive language samples from all compliant participants with ASD**										
	**NSSP group (*****n*** **=** **9)**					**NESP group (*****n*** **=** **8)**				
	**Paired samples statistics**		**Paired samples test** **Paired Differences**			**Paired samples statistics**		**Paired samples test** **Paired differences**		
	**Test**	**Retest**				**Test**	**Retest**			
	**Mean (SD)**	**Mean (SD)**	**Mean (SD)**	**95% CI**	***T***	**Mean (SD)**	**Mean (SD)**	**Mean (SD)**	**95% CI**	***t***
Lexical diversity	95.9 (61.4)	99.6 (57.6)	−3.8 (14.9)	[−15.2, 7.7]	−0.8	89.6 (29.2)	91.8 (32)	−2.3 (8.6)	[−9.5, 5]	−0.7
Syntactic complexity	7.3 (3.7)	7.2 (3.4)	0.16 (0.8)	[−0.5, 0.8]	0.6	7 (1.7)	6.5 (1.7)	0.5(0.8)	[–.02, 1.2]	1.6
Talkativeness	8 (1.5)	8.7 (2.7)	−0.67 (1.9)	[−2.1, 0.8]	−1.1	8.7 (1.3)	10.1 (2.9)	−1.5 (2)	[−3.1, 0.2]	−2.1
Intelligibly	0.2 (0.3)	0.2 (0.2)	0.07 (0.1)	[0.001, 0.2]	2.4^*^	0.03 (0.1)	0.08 (0.1)	−0.04 (0.1)	[−0.1, 0.01]	−2
Dysfluency	0.3 (0.2)	0.3 (0.2)	−0.01 (0.1)	[−0.1, 0.1]	−0.4	0.3 (0.2)	0.3 (0.1)	0.02 (0.1)	[−0.1, 0.1]	0.5
**Expressive language samples from compliant participants with ASD whose caregivers administered the ELS-N at or above an 80% of fidelity**										
	**NSSP group (*****n*** **=** **7)**					**NESP group (*****n*** **=** **5)**				
	**Paired samples statistics**		**Paired samples test** **Paired Differences**			**Paired samples statistics**		**Paired samples test** **Paired differences**		
	**Test**	**Retest**				**Test**	**Retest**			
	**Mean (SD)**	**Mean (SD)**	**Mean (SD)**	**95% CI**	***T***	**Mean (SD)**	**Mean (SD)**	**Mean (SD)**	**95% CI**	***t***
Lexical diversity	109.3 (63.8)	111.6 (60.7)	−2.3 (16.5)	[−17.5, 12.9]	−0.4	96.6 (22.2)	100.6 (29.1)	−4 (9.6)	[−15.9, 7.9]	−0.9
Syntactic Complexity	7.5 (4.1)	7.4 (3.6)	0.1 (0.8)	[−0.7, 0.8]	0.2	7.4 (1.1)	7.2 (1.3)	0.2 (0.8)	[−0.8, 1.3]	0.6
Talkativeness	8.6 (1.2)	9.2 (2.9)	−0.6 (2.1)	[−2.5, 1.3]	−0.8	8.6 (1.7)	9.8 (3.6)	−1.2 (2.4)	[−4.2, 1.8]	−1.1
Intelligibly	0.2 (0.3)	0.2 (0.2)	0.1 (0.1)	[−0.04, 0.1]	1.5	0.01 (0.02)	0.03 (0.03)	−0.01 (0.02)	[−0.03, 0.01]	−1.8
Dysfluency	0.3 (0.2)	0.3 (0.2)	0.01 (0.1)	[−0.1, 0.1]	0.3	0.4 (0.2)	0.3 (0.1)	0.04 (0.1)	[−0.1, 0.2]	0.8

*The table presents t-tests (t) for correlated samples to determine whether differences in means from test to retest of the ELS-N for each of the five language variables were significant (practice effects) for each language group (NNSP vs. NESP). None of the t-tests comparing the first and retest administrations was significant at p < 0.050 (even uncorrected for multiple tests) for any of the subgroups. NSSP, native Spanish-speaking parents; NESP, native English-speaking parents; SD, standard deviation; CI, confidence interval. Note that uncorrected p-values for individual tests are marked with asterisk as follows:*

* *p ≤ 0.050*.

As observed in Table 5, no significant differences (*p* < 0.05) were observed in scores, for any of the 5 language variables, across the test and retest administrations. Thus, practice effects were not observed for either subgroup, regardless of the parent primary language, or whether the parent had maintained fidelity at both the test and retest administrations (see Table 5 for details).

Table 6 shows data regarding test-retest reliability. As observed, for the NSSP group, all bivariate and intraclass correlations for all language variables were significant (regardless of parent achieving fidelity) before and after correcting for multiple tests, except for *talkativeness*. For the NESP group, it can be seen in Table 6 that all bivariate and intraclass correlations were significant before correcting for multiple testing; however, only the correlations for *lexical diversity* and *syntactic complexity* remained significant after the Bonferroni correction, with the latter only significant in the full sample analysis. Note, however, the smaller sample sizes for the NESP group and that many of the correlations were large in absolute magnitude.

**Table 6:** Test-retest reliability over a 4-week interval: bivariate correlations and intraclass correlations.

**Expressive language samples from all compliant participants with ASD**				
	**NSSP group (*****n*** **=** **9)**		**NESP group (n=8)**	
	**Test-retest Statistics**		**Test-retest Statistics**	
	**Pearson correlation (r)**	**Intraclass correlation (icc)**	**Pearson correlation (r)**	**Intraclass correlation (icc)**
Lexical diversity	0.97^***^	0.97^***^	0.96^***^	0.96^***^
Syntactic Complexity	0.98^***^	0.98^***^	0.88^**^	0.86^**^
Talkativeness	0.76^*^	0.64^*^	0.83^*^	0.54^*^
Intelligibly	0.94^***^	0.87^***^	0.80^*^	0.72^*^
Dysfluency	0.89^***^	0.90^***^	0.76^*^	0.75^*^
**Expressive language samples from compliant participants with ASD whose caregivers administered the ELS-N at or above an 80% of fidelity**				
	**NSSP group (*****n*** **=** **7)**		**NESP group (n** **=** **5)**	
	**Test-retest Statistics**		**Test-retest Statistics**	
	**Pearson correlation (r)**	**Intraclass correlation (icc)**	**Pearson correlation (r)**	**Intraclass correlation (icc)**
Lexical diversity	0.97^***^	0.97^***^	0.97^**^	0.93^**^
Syntactic Complexity	0.99^***^	0.98^***^	0.76^*^	0.77^*^
Talkativeness	0.82	0.59	0.83	0.63
Intelligibly	0.95^**^	0.91^**^	0.85^*^	0.73^*^
Dysfluency	0.92^**^	0.93^**^	0.73	0.72

*The table presents Pearson correlations (r) and intraclass correlations (icc) between the test and retest administration of the ELS-N for each of the five language variables. Note that uncorrected p-values for individual tests are marked with asterisks as follows:*

*** *p ≤ 0.0005;*

** *p ≤ 0.005;*

* *p ≤ 0.050*.

*Bolded values correspond to those that were significant after Bonferroni correction (p < 0.005). Abbreviations: NSSP, native Spanish-speaking parents; NESP; native English-speaking parents*.

### What Are Parental Opinions of the Training and the ELS-N Task?

In terms of parental descriptions of the adequacy and acceptability of training and comfort administering the procedures within the context of a clinical trial, we observed the following: First, 18 of the 19 parents who completed training reported that the task was easy to learn, and they were confident that they learned to administer it correctly. The one mother who thought she had not learned to administer the ELS-N task was from the NSSP group and her perception was consistent with her fidelity data (Table 3; ID: NSSP-1). In addition, all 19 parents reported that the training was adequate and that they felt comfortable with it. Regarding the language and cultural appropriateness of the learning materials and the ELS-N task, all the parents from the NSSP group reported that the training instructions and feedback as well as the books and scripts used during the narrative task were appropriate in terms of language and culture. However, regarding the books, one NSSP reported that these might not be appropriate for older children, another NSSP reported that the books were a bit boring, and one NESP suggested that the books were too long. In addition, three parents from the NSSP reported that the training was difficult for them in terms of scheduling, and two parents from the NSSP group stated that the technology procedures used in the training were demanding but still feasible to master. Finally, 12 (i.e., 5 NSSP and 7 NESP) of the 19 (i.e., 10 NSSP, 9 NESP) parents who completed training reported that if they were in a clinical trial, they would prefer to administer the task at home rather than at a clinic, 5 parents (i.e., 4 NSSP and 1 NESP) reported that they would prefer to bring their children to the clinic for an examiner administration rather than administer the task at home, and two parents (i.e., 1 NSSP and 1 NESP) reported no preferences. The main reasons why parents preferred to administer the task at home were related to convenience (e.g., “We don't have to travel to the clinic, which is great since we live in a rural area”) and behavioral reasons (e.g., “If my son has a bad day, I can reschedule the task for another moment”). Finally, the reasons why parents preferred to bring their child to the clinic were: (1) parents reported feeling more confident if a clinician evaluates the child (e.g., “I think assessments need to be done by professionals,” “It's hard not to over prompt my child, so I feel more confident if a professional gives the task”) and –) due to behavioral reasons (e.g.,” I feel like my child takes it more seriously if he's evaluated by a stranger”).

## Discussion

The goal of the present pilot study was to obtain preliminary data on the feasibility of the home-based parent-implement ELS-N procedures for English- and Spanish-speaking children and adolescents with ASD residing in the U.S.

Our results indicate that most of the parents who participated in this project were able to learn to administer the ELS-N procedures to their children at an appropriate level of fidelity when the training was given in their primary language. In addition, parents were able to handle the technology-related procedures needed for the implementation of the task across the multiple ELS-N administrations, even if not using their own technology (which was the great majority of the families). In this regard, only two parents reported that the technology used in the training was demanding but still feasible to master. These findings support the promise of the procedures for clinical trials. At the same time, however, more research is needed to determine whether additional tools or training are required for parents to maintain fidelity standards over an extended period of time. These data will be important considering that longitudinal treatment studies may require several ELS-N administrations over months or even years. In this regard, note that 3 of the 16 parents who achieved fidelity at the test session, dropped below the target fidelity standard at the retest administration a month later. These data suggest that at least some parents may need additional support even in short-term clinical trials.

Regarding the extent to which the participants with ASD were able to complete the narrative procedures in meaningful ways, data from the current study is limited by the small sample size. However, results suggest that the procedures are feasible for many 6- to 21-year-olds with ASD, which is in line to what has been found for the original ELS-N procedures administered by professional examiners (26, 27). Therefore, participants with ASD in this study were largely successful in producing complete narrations, and that was true regardless of the language used during testing. Nonetheless, and line with previous research (26), task compliance was related to concrete participant's characteristics, such as the level of expressive language maturity (in this study reported by parents). Interestingly, when comparing mean scores on the *syntactic complexity* and the *lexical diversity* of the participants from the current study to the mean scores of typical developing (TD) participants with the same mean age (10.5) from the Channell et al. (20) study, we see that the mean length of an utterance (i.e., C-unit) in morphemes (i.e., syntactic complexity) is 2.5 morphemes less in the current study (Channell study *M* = 9.5, current study *M* = 7). In addition, the mean number of different word roots in up to 50 C-units (i.e., lexical diversity) is around 17-word roots lower in the current study compared to the TD participants from the Channell study (Channell study M = 110, current study M = 93.2). These comparisons suggest that, at a group level, participants with ASD from the current study showed lower expressive language skills than what would be expected based on their chronological age, which is consistent with the expectation for this population based on previous research (32). These comparisons, however, should be interpreted cautiously because the Channell et al. data are based on the original ELS-N procedures administered by professionals to English-speaking participants. Further studies are needed to validate the home-based parent-implemented ELS-N procedures in TD participants within the same age range and primary language as in the current study in order to conduct reliable comparisons.

Regarding the extent to which variables derived from this parent-administered version of the ELS-N procedure are psychometrically sound, our results suggest that the language variables appeared to be subject to minimal practice effects while also displaying strong test-retest reliability regardless of the language of testing. Therefore, our results are consistent with results in previous research on the psychometric proprieties of the ELS-N methods administered by professional examiners to English-speaking children and adolescents with other neurodevelopmental conditions in a clinic setting, which have shown that the task has no practice effects and good test-retest reliability (26, 27). In addition, the psychometric properties of the measures derived from the current study were adequate even when we included in the analyses those parent-child dyads whose parents did not administer the ELS-N at the target level of fidelity. This latter finding suggests that perhaps less rigorous training and fidelity standards could be adopted for clinical trials than required in this study. At the same time, it may be that certain core elements of the administration are critical for the assessment, whereas others may be less important, at least in terms of influencing the expressive language of the youth. Finally, although the studies of the professional-administered version of the ELS-N in English have documented strong construct validity for several of the measures, at least for individuals with DS (27) or FXS (26), discriminant and convergent validity of the parent-implemented version of the ELS-N procedures was not evaluated in this study. Thus, whether each language measure derived from the ELS-N when implemented by parents correlates with the intended language construct remains unknown.

Regarding the parental opinions on the training, one key finding is that 63% of the parents reported that they would prefer to administer the task to their sons and daughters at home in lieu of bringing them to the clinic and having a professional administer the task. Only 26% reported that they would prefer to bring their child to the clinic, with the remaining 11% reporting no preferences. These results are of particular interest given that most clinical trials require multiple administrations of the outcome measures and, thus, many trips to the clinic. This can be a burden that is costly to the family, discouraging many families from enrolling and contributing to a higher attrition rate. The requirement of frequent travel is particularly problematic in the case of many individuals with ASD because they can have co-occurring problems such as anxiety and challenging behaviors (e.g., aggression in response to disruptions in routine). At the same time, however, other families may feel more comfortable bringing their child to the clinic and having a professional administer the task. Therefore, rather than being an “all or of none” approach, having some families coming for in-person visits and some families to complete the administrations themselves based on their preferences may not only promote the enrollment of several families that otherwise would not be able to participate but also will help to ensure more diverse and representative samples in clinical trials on ASD. Also, note that current options for gathering data in the home typically entail parent completion of various questionnaires and scales about child behavior, which have been found to be subject to large placebo effects (39). The ELS-N procedures, when administered by the parent at fidelity, should avoid the problem of placebo effects.

An important contribution of the present study is that by adapting the ELS-N procedures to Spanish, we are also ensuring the inclusion of participants with ASD who speak Spanish. In light of the growing Spanish-speaking population in the U.S., the lack of measures validated for Spanish participants leads to an increasingly larger proportion of U.S. citizens being ineligible for clinical trials, which again, limits the generalizability of results and effectively prevents them from accessing the potential benefit of the treatment being investigated. In addition, we want to make a few points about the lessons learned from the NSSP group and the future directions. First, we tried to create a Spanish version of the ELS-N task, as well as a training protocol for parents, that were linguistically and culturally appropriate. The findings, including parent responses in the exit interviews, suggest that we were successful in this regard. Second, the Spanish-speaking families in our sample, like many families in the U.S., were variable in their language preferences and degree of bilingualism of both the parents and the youths. Note that despite all parents from the NSSP group were trained in Spanish, some parents chose to administer the task entirely in English (given the fact that their children were monolingual English-speakers) and some other parents combined verbal productions in Spanish and English when administering the task. This data not only suggests that these parents felt confident enough about the ELS-N procedures to be flexible in their administrations, but also the need of that language flexibility in their ELS-N administrations.

### Suggestions for Bilingual Language Sampling

Given the observed language flexibility in completing the ELS-N procedures by the NSSP, authors suggest that language assessments, whether for clinical purposes or research, should carefully consider family preferences and practices when deciding on the language/s of assessment, and this should be the case whether the tester is the parent or a professional. More generally, it will be increasingly necessary to take a more dynamic approach to language sampling, using language practices of bilingual people as the norm in terms of both administration and interpretation [i.e., *translanguaging* by Otheguy et al. (40)]. More in detail, *translanguaging* is an alternative sociocultural understanding of bilingualism described as the process whereby multilingual speakers use their languages as an integrated communication system (41). Adopting such approach would entail, among other things, allowing the child to shift between languages when speaking and to interact with a bilingual examiner who can also match the child's within-sample language shifts so that the child feels comfortable and fully supported.

In following the suggested approach, the authors have recently published a case-report about a family of a father and a mother and two boys with ASD for the sample in the present study. The selected family in which parents were bilingual native Spanish-speaker and participants with ASD monolingual English-speakers (38). The flexibility adopted for this family–for whom the training was provided in Spanish, but the task was given to the child in English (e.g., during the coaching session we guided the parent in Spanish but indicated the prompts to be used on the child in English)–was successful and authors believe that future studies about parent implemented interventions or assessments must follow this approach for those families in the same situation [see del Hoyo Soriano et al. (38) for details on this case report].

### Limitations and Future Directions

Several limitations should be considered when interpreting results of the current study. First, it is important to point out that the entire training process for each parent lasted a total of 9–10 weeks. This duration of training could be a burden for many families implementing the ELS-N procedures in the context of a treatment study, particularly when this training time is added to the time commitment required by the treatment study/clinical trial itself. Thus, there is a need to determine whether a less intense training can yield language samples that are representative of the youths' language skills and the variables derived psychometrically sound. Second, the small sample size tempers our conclusions with regards to the psychometric proprieties of the ELS-N procedures when administered by parents from home. Third, information regarding the clinical characteristics of the participants with ASD (including their cognitive and functional levels) was limited to what was provided by caregivers in the eligibility interview (e.g., all participants had non-syndromic ASD, they were verbal, and they were living at home with caretakers who were making clinical decisions on their behalf). Future larger samples are needed in which participant's cognitive and functional skills as well as expressive language abilities are evaluated through standardized measures. Fourth, future studies will also need to focus on comparing the psychometrics of the professional-administered ELS-N vs. the parent-implement ELS-N, which will be useful for deciding on the administration mode best for any given clinical trial. Additionally, further studies with larger samples are also needed to validate the Spanish version of the ELS-N as well as a dual-language implementation (i.e., Spanish/English) of the same. Finally, and based on the previous research on the ELS-N, we plan to extend the home-based parent-implemented ELS-N procedures to other neurodevelopmental conditions, such as DS and FXS, as well as to TD participants.

## Conclusions

In conclusion, the findings reported in the current pilot study show that most of the English- and Spanish-speaking parents who participated in this project were able to learn the ELS-N procedures in their native language to the target level of fidelity, with the procedures being feasible for the majority of the 6- to 21-year-old participants with ASD, and with no practice effects and appropriate test-retest reliability. One should keep in mind that this is the first time that the ELS-N has been implemented by parents from home, and the first time that the ELS-N procedures have been adapted to Spanish. Therefore, this small-scale pilot project has served as a first step, providing preliminary data of the promise of the approach and the value of additional research.

## Data Availability Statement

The datasets used and/or analyzed during the current study as well as the materials used for the parent training are available from the corresponding author upon reasonable request.

## Ethics Statement

The study was approved by the Institutional Review Board of the University of California, Davis. Written informed consent from all parents and verbal assent from all participants with ASD was obtained before their participation in the study.

## Author Contributions

LHS conceived the overall design of the study and protocol, designed the parent training materials and the Spanish version of all the ELS-N materials, conducted the focus group, led the parent training, conducted the analytical plan, collected and analyzed the data, interpreted the results, and wrote the manuscript. LA conceived the overall design of the study and protocol oversaw data collection, contributed to the analytical plan for this study, and the interpretation of the results. AT was responsible for constructing and validating the language sampling data sets and assisted with interpretation of the findings. LB conducted the training of the English-Speaking families, designed the telehealth-related procedures, and assisted with interpretation of the findings. CH led the transcription of the language samples from the NSSP group, contributed to the validation of the training materials and assisted with interpretation of the findings. All authors reviewed, edited, and approved the manuscript.

## Funding

This study was funded by the National Institutes of Health through the UC Davis Clinical and Translational Science Center (UL1-T001860) and through grants R01HD074346 and P50HD103526.

## Conflict of Interest

LA has received funding from F. Hoffmann-La Roche Ltd., Roche TCRC, Inc., Neuren Pharmaceuticals Ltd., Azrieli Foundation, and Lumind to consult on and implement outcome measures in clinical trials for FXS and DS. AT has received funding for the development and implementation of treatment outcome measures from Fulcrum Therapeutics and the Azrieli Foundation. The remaining authors declare that the research was conducted in the absence of any commercial or financial relationships that could be construed as a potential conflict of interest.

## Publisher's Note

All claims expressed in this article are solely those of the authors and do not necessarily represent those of their affiliated organizations, or those of the publisher, the editors and the reviewers. Any product that may be evaluated in this article, or claim that may be made by its manufacturer, is not guaranteed or endorsed by the publisher.

## Footnotes

^1^Note that families participating in this project did it between March of 2019 and February of 2021. For those parents to whom this question was asked from March 2020 to February 2021, the specific question was reformulated as whether they would prefer to administering the task to their children at home in lieu of bringing them to the clinic, if there wasn't a pandemic.
